# The effect of teamwork training on team performance and clinical outcome in elective orthopaedic surgery: a controlled interrupted time series study

**DOI:** 10.1136/bmjopen-2014-006216

**Published:** 2015-04-18

**Authors:** Lauren Morgan, Mohammed Hadi, Sharon Pickering, Eleanor Robertson, Damian Griffin, Gary Collins, Oliver Rivero-Arias, Ken Catchpole, Peter McCulloch, Steve New

**Affiliations:** 1 Nuffield Department of Surgical Sciences, University of Oxford, Oxford, UK; 2 Warwick Medical School, University of Coventry and Warwick, Warwick, UK; 3 Centre for Statistics in Medicine, University of Oxford, Oxford, UK; 4 Nuffield Department of Population Health, University of Oxford, Oxford, UK; 5 Red de Investigación de Servicios Sanitarios en Cronicidad (REDISSEC), Spain; 6 Cedars-Sinai Medical Centre, Los Angeles, California, USA; 7 Saïd Business School, University of Oxford, Oxford, UK

**Keywords:** SURGERY, Quality improvement, Patient safety

## Abstract

**Objectives:**

To evaluate the effectiveness of aviation-style teamwork training in improving operating theatre team performance and clinical outcomes.

**Setting:**

3 operating theatres in a UK district general hospital, 1 acting as a control group and the other 2 as the intervention group.

**Participants:**

72 operations (37 intervention, 35 control) were observed in full by 2 trained observers during two 3-month observation periods, before and after the intervention period.

**Interventions:**

A 1-day teamwork training course for all staff, followed by 6 weeks of weekly in-service coaching to embed learning.

**Primary and secondary outcome measures:**

We measured team non-technical skills using Oxford NOTECHS II, (evaluating the whole team and the surgical, anaesthetic and nursing subteams, and evaluated technical performance using the Glitch count. We evaluated compliance with the WHO checklist by recording whether time-out (T/O) and sign-out (S/O) were attempted, and whether T/O was fully complied with. We recorded complications, re-admissions and duration of hospital stay using hospital administrative data. We compared the before–after change in the intervention and control groups using 2-way analysis of variance (ANOVA) and regression modelling.

**Results:**

Mean NOTECHS II score increased significantly from 71.6 to 75.4 in the active group but remained static in the control group (p=0.047). Among staff subgroups, the nursing score increased significantly (p=0.006), but the anaesthetic and surgical scores did not. The attempt rate for WHO T/O procedures increased significantly in both active and control groups, but full compliance with T/O improved only in the active group (p=0.003). Mean glitch rate was unchanged in the control group but increased significantly (7.2–10.2/h, p=0.002) in the active group.

**Conclusions:**

Teamwork training was associated with improved non-technical skills in theatre teams but also with a rise in operative glitches.

**Strengths and limitations of this study**

- Standardised, well-validated semiobjective measures.
- Before–after design with parallel control group.
- Continuous observation of whole procedures by paired expert observers.
- Difficulty in implementing training intervention.
- Improvement in non-technical skills but deterioration in technical performance of trained teams.

## Introduction

The reliability of operating theatre teamwork and its role in ensuring error-free surgery, thereby reducing the risks of harm to patients, has been studied intensively in recent years. Problems of miscommunication and poor teamwork associated with hierarchy, fatigue and stress from dysfunctional relationships between professional groups have been reported.^1–4^ Minor technical errors and deviations from intended practice have been shown to be commonplace, and there is evidence that operations with a large number of these are more likely to suffer a serious error or mishap with real or potential harm to the patient.^5^ Evaluation of the causes of error and harm in operating theatres has highlighted the importance of the interaction between team members and specifically their ‘non-technical skills’ in relating to and communicating with each other. This field of study has been informed by a body of work in civil aviation, linking the safety of airlines to their crew culture.^2^ Principles for improving teamwork have been formalised in civil aviation in mandatory ‘Crew Resource management’ (CRM) training, which is widely credited with improving the safety of flying. Parallels between aviation and surgery have been made on the basis of which CRM training has been proposed as a means of improving safety and reliability in operating theatres.^6^ There is now a significant amount of literature on this subject,^7^ but the majority of studies are either artificial and short term or uncontrolled, with a high risk of bias from secular trends and Hawthorne effects. Our own previous work showed general approval of the training, improved safety attitudes, improved non-technical skills and reduced technical error rates, thus providing convincing evidence of benefit according to the Kirkpatrick model for training evaluation.^8^ ^9^ However, this study was also uncontrolled, and we observed a rapid fall-off in effect once support for the team was withdrawn. The measures used were also recognised as imperfect and have subsequently been revised.^10^ ^11^ We therefore felt it was important to repeat the study in a controlled experiment using our revised measures. We conducted this study in the context of a larger research programme which focused on the relative merits of interventions to improve systems, culture or both in reducing harm in clinical settings.

## Methods

### Setting

We studied staff in two dedicated elective orthopaedic theatres (the intervention group) and a vascular/general surgery theatre (the control group) in the main operating suite of a District General Hospital. The main operations performed were hip and knee replacements in the intervention group and varicose vein surgery, femoro-popliteal artery bypass and inguinal hernia repair in the control group. An orthopaedic control group was not possible due to the small and compact orthopaedic unit in this Trust,^i^ which had a high degree of staff interchange between theatres.

### Study design

The study was designed as an interrupted time series with observations made for 3 months before and 3 months after a 3-month intervention period. Contemporaneous observations in the control group were made without any intervening intervention.

### Intervention and manner of delivery

The intervention was a course of teamwork and communications training based closely on aviation CRM as developed by an external aviation consultancy (Atrainability). The course consisted of two 3 h sessions of interactive classroom teaching delivered by retired civil aviation pilots who had an extensive background in CRM training for aircrew, and several years’ experience of adapting this to train theatre staff. Specific attention was given to the relevance of the training to the performance of the WHO surgical checklist. After completing classroom training, the trainers returned regularly to provide on-the-job coaching to each theatre over the next 6 weeks. We attempted to give training to all members of the surgical, nursing and anaesthetic staff who regularly worked in the intervention group theatres. We provided several opportunities to attend, and negotiated free time and staff back-fill with management, as well as publicising the training in a number of different ways. In preliminary discussions, we attempted to gain the engagement of the consultant surgeons and anaesthetists, theatre team leaders and theatre and surgical managers. We held meetings with theatre nursing staff to explain the ideas behind the training, to reassure them and to answer questions.

### Measures

We assessed the process effects of the intervention with three measures: Non-technical Skills team assessment using Oxford NOTECHS II, a scale developed during this programme and based on our previous work;^8^ ^12^ a count of operative process glitches; and evaluation of WHO checklist completion. We evaluated patient outcomes using HES (Hospital Episode Statistics) data from the hospital administrative system on length of stay, complications and re-admissions within 90 days for all patients operated on during the relevant theatre lists during the 6 months before and the same period after the intervention. The Oxford NOTECHS II behavioural rating scale scores each subteam: (nursing, surgical and anaesthetic) on a 1–8 scale against four behavioural parameters: Leadership and Management; Teamwork and Cooperation; Problem solving and Decision making; and Situational Awareness. Summing the subteam scores gives an optimum score of 96 for a perfectly performing team. Technical performance was evaluated by counting glitches, defined as deviations from the recognised process with the potential to reduce quality or speed, including interruptions, omissions and changes, whether or not these actually affected the outcome of the procedure.^10^ Glitches were counted independently by each observer noting the time and details of the glitch (eg, ‘diathermy not plugged in when surgeon trying to use it’) in standardised data collection booklets.^13^ Glitches were subsequently agreed by observers, categorised and entered into a secure database. A glitch rate per hour was calculated, allowing operations of differing lengths to be compared. To evaluate WHO Surgical Safety Checklist performance, data was collected on whether the time-out (T/O) and sign-out (S/O) sections of the checklist were attempted. Observers also recorded three measures of process quality: (1) whether all the specified information was communicated, (2) whether all the team was present and (3) whether they judged that the team showed active participation.^14^

Hospital episode statistics data were extracted for all patients undergoing operations in the relevant operating theatres under the involved consultants during the 6-month periods immediately before and after the intervention. This, therefore, represents a larger group of patients, of which those whose operations were observed represented a large convenience sample. Data were independently extracted by the Trust staff and supplied to the research team in anonymised form. The information extracted for each patient was: age, sex, diagnosis, consultant, operation, operating time, length of hospital stay, complications (if any) and nature, readmission within 90 days of operation/reoperation. The parameters used in comparisons between active and control groups were: length of stay, number (%) of patients with any complication and readmissions within 90 days.

### Manner of collecting the data

Each operation was observed by two observers; one with a clinical and the other with a human factors (HF) background. The clinical observers included two surgical trainees (MH, ER) and one nurse practitioner, the HF specialists had a higher degree in HF and/or psychology (SP, LM). Before the study, observers completed a 2-month training phase for familiarisation with surgical procedures and to agree and harmonise norms on how to record events. Intraoperative observation began when the patient entered the theatre, and ended when they left the operating theatre.

### Data analysis

The difference between the control and active arms was assessed using two-way analysis of variance (Group×Time), with treatment (control vs active) and time (preintervention vs postintervention) as factors. Differences between groups were assessed by the Group×Time interaction. Preintervention and postintervention differences are reported as 95% CIs. All statistical analyses were carried out in R (V.3.0.1). For clinical outcome data, t tests for mean age and χ^2^ test for gender distribution were used to compare the before and after periods. Binary clinical outcome variables before and after intervention were compared using ORs and 95% CIs from a logistic regression, and mean length of stay using linear regression, controlling for age and gender in both regression models. Given the number of before and after comparisons performed, a 1% significance level was selected. This analysis was conducted in Stata V.12.

### Ethics

Patients whose operations were observed were informed of the possibility of observations taking place, and were given the opportunity to opt out if they wished. Staff in the theatres undergoing observation were given information on the study and asked for consent before observations took place.

## Results

Twenty-six operations were studied before the intervention in the active theatres and 11 in the control theatres, compared with 25 and 10 operations, respectively, after the intervention. The types of surgery performed remained stable throughout in both groups. The average operating time reduced from 1 h 38 min to 1 h 11 min in the control group but remained static at about 1 h 55 min in the active group. There was no significant change in the mean patient age or the gender balance in either group (table 1).

**Table 1:** Characteristics of patients and procedures before and after intervention

	Active before	Active after	Control before	Control after
Mean age (years) of the patient	57.8	57	51.7	52.2
Male patients (%)	48	48	52	51
Mean duration of operation in minutes (range)	113 (61–235)	115 (73–219)	98 (30–144)	71 (30–140)
Performed by consultant, %	49	51	59	41

## Oxford NOTECHS II

**Figure 1:**
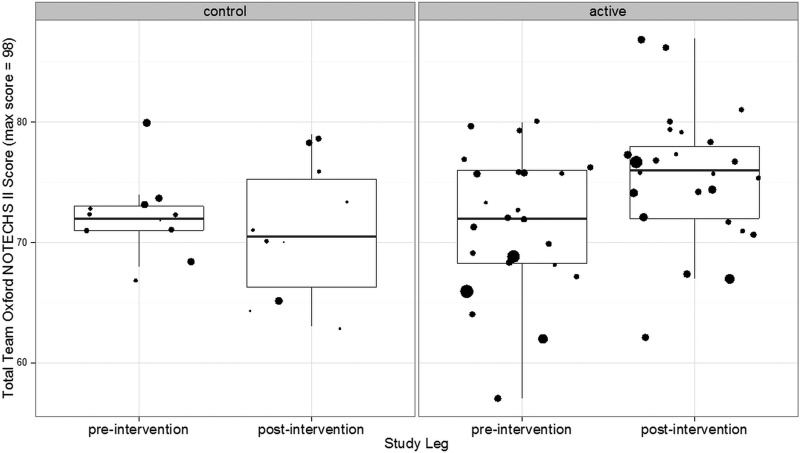
Oxford NOTECH II scores for all operations before and after interventions. The size of spots relates to the duration of operations. The box and whisker plots delineate the median and IQR.

Mean NOTECHS score increased from 71.62 before to 75.44 after the intervention in the active group (difference 3.82; 95% CI 0.67 to 6.98), while it remained unchanged in the control group (72.09 before, 70.09 after, difference=−1.19; 95% CI −5.62 to 3.24). The difference between the change in the active and control groups was statistically significant (p=0.047; difference 4.54; 95% CI 0.06 to 9.02). Subteam analysis revealed that differences in mean NOTECHS scores were non-significant for surgeons (p=0.806) and anaesthetists (p=0.067), while statistically significant for nurses (p=0.006; difference 1.78; 95% CI 0.40 to 3.16) (figure 1).

### WHO compliance

T/O was attempted in 51 of the 72 observed operations. The T/O attempt rate improved significantly in the active group (preintervention 11/26; 42%, postintervention 25/25; 100%, difference=58%; 95% CI −35% to 81%; p<0.001), but also in the control group (preintervention 5/11; 45%, postintervention 10/10; 100%, difference=55%; 95% CI 16% to 94%; p<0.001). There was no significant difference between the degree of improvement between the active and control groups (p=0.640). All three components of T/O were completed in 4/26 (15%) cases in the preintervention active arm, which increased to 23/25 (92%) in the postintervention phase (difference=77%; 95% CI 55% to 98% p<0.001). All three components of T/O were completed in 0/11 (0%) cases in the preintervention control arm, which increased to 2/10 (20%) in the postintervention phase (difference=20%; 95% CI −14% to 54%; p=0415). The increase in compliance was significantly better in the CRM group (p=0.003).

**Figure 2:**
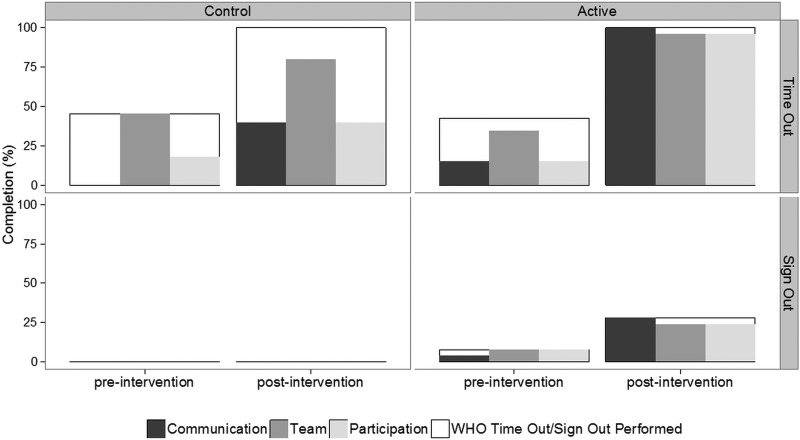
Quality of WHO checklist completion.

S/O was attempted in only 9 of the 72 observed operations. There was a small difference in the attempt rate of S/O between preintervention (2/26; 8%) and postintervention (7/25; 28%) in the active arm (difference=20%; 95% CI −4% to 45%; p=0.125), but no difference between the preintervention (0/11; 0%) and postintervention (0/10; 0%) in the control arm. The difference between the changes in the active and control groups was not significant (difference 28%; 95% CI 4% to 53%; p=0.161). The quality of WHO completion is compared preintervention and postintervention for control and active groups in figure 2. The marked improvement in the active group was significant when compared with the control group (p<0.05).

### Glitch count

**Figure 3:**
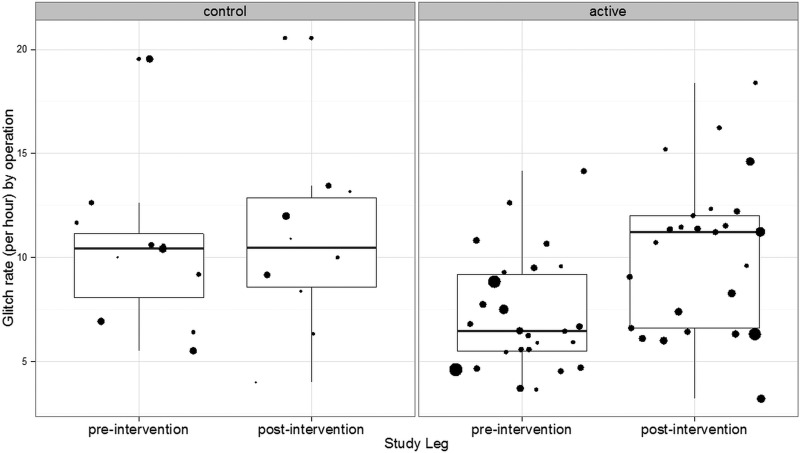
The profile of glitches encountered across the control/active preoperative/postoperative phases.

The mean glitch rate per operation was 7.21 (±SD 2.73) glitches per hour in the active group and 10.31 (±3.79) in the control group before the intervention (figure 3). After the intervention, the mean glitch rate increased significantly to 10.20 (±3.67) in the active group (difference=2.99; 95% CI 1.16 to 4.82; p=0.002) while it remained essentially unchanged in the control group (10.79±4.53) postintervention (difference 0.48 95% CI −3.38 to 4.34; p=0.796). The difference between the changes in the active and control groups was not statistically significant (p=0.173).

### Clinical outcomes

There was a rise in the complication rate in the active group after the intervention and a small fall in the rate in the control group: the difference between these two just reached significance (p=0.05, table 2). There were minor changes in readmission rates and length of stay in both groups, but neither difference reached significance, and the trends were in opposite directions.

**Table 2:** Summary outcome measures

	Control		Intervention		
	Preintervention	Postintervention	Preintervention	Postintervention	p Value
NOTECHS	72.09 (3.36)	70.09 (5.70)	71.62 (5.69)	75.44 (5.53)	0.047
WHO T/O attempted, n (%)	5/11 (45)	10/10 (100)	11/26 (42)	25/25 (100)	0.640
WHO T/O success, n (%)	0/11 (0)	2/10 (20)	4/26 (15)	23/25 (92)	<0.05
WHO S/O attempted, n (%)	0/11 (0)	0/10 (0)	2/26 (8)	7/25 (28)	0.125
Glitch rate/h	10.31 (3.79)	10.79 (4.53)	7.21 (2.73)	10.20 (3.67)	0.173
Complication rate, n (%)	162 (27.1)	106 (25.7)	140 (21.5)	185 (26.8)	0.05
Length of stay (days)	4.82 (13.5)	4.93 (11.7)	5.09 (11.1)	5.38 (13.2)	0.371
Readmission rate, n (%)	51 (8.5)	37 (9.0)	72 (13)	74 (11)	0.25

S/O, sign-out; T/O, time-out.

## Discussion

**Figure 4:**
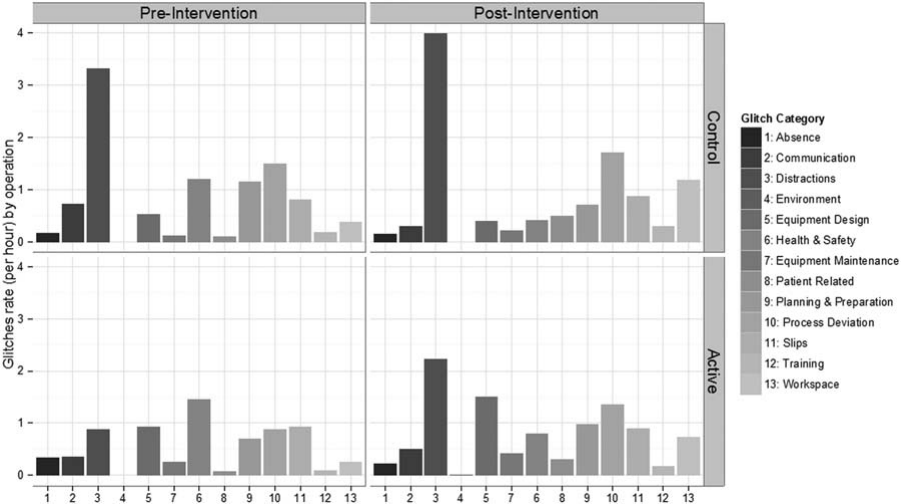
Mean glitch rate by operation for active and control arms, preoperative and postoperative. Figures are mean (SD) unless otherwise specified.

In this study, we found that teamwork training was associated with improved team non-technical skills, but that technical performance declined and complications rose marginally compared with the control group. Compliance with the WHO checklist procedures rose significantly, but it rose equally in the control group who did not receive training. Interpretation of these inconsistent results requires attention to the specific challenges and circumstances of this study. Implementation of training was beset by a variety of logistic and organisational difficulties which may have detracted from its effectiveness. Attendance of both nursing and surgical staff at training proved very difficult to arrange due to communication errors, prioritisation of clinical activities over cooperation with training, divisions within the Trust of which we were not made aware, and reluctance to risk performance targets by reducing activity to allow training. Ultimately however, training was delivered to most of the target staff group, and was significantly associated with increased non-technical skills, its primary target. The finding that the nursing NOTECHS scores were almost entirely responsible for the post-training rise is consistent with our findings from previous studies using CRM-type training^8^ and with those of others.^15^ The overall rise of over four points was not only statistically significant but functionally important, as the range of scores expected with this tool is quite restricted.^11^ We were unable to account for the worsening glitch rate in the active group, although the glitch category data (figure 4) suggest that training actually increased distractions substantially. This is unfortunately not likely to be due to an increase in ‘speaking up’ about problems, as the observers were trained to score this type of intervention positively, and were unanimous in agreeing that such behaviour would not be scored as a distraction. The increase in complications found in this group is consistent with theory, and with previous work linking small technical imperfections with risks of harm to patients.^5^ Our validation work on the glitch rate measure^10^ has demonstrated that it does not correlate with NOTECHS II, and we postulated that this may be because ‘glitchy’ operations may provide opportunities for teams to demonstrate superior non-technical skills, while poor non-technical skills might increase glitch rates, so the relationship between the two measures is complex and non-linear. No changes in team makeup, activity or morale could be identified to provide alternative explanations for the rise in glitches we observed. The environment glitch category (which includes problems with heating, noise, structural integrity, power and lighting, computer function, other environmental features) could not have explained the changes seen, since it normally represents a small percentage (about 3%) of all glitches,^10^ and by chance, none occurred in the relatively small sample in this study. Our previous studies of CRM training showed a positive effect on technical performance,^8^ but this used a substantially different taxonomy which showed a clear correlation with measures of non-technical skills.

The unexpected rise in WHO T/O compliance in the control group, and the unexpected rise in glitches in the active group after intervention, prompt reflection on the limitations of our direct observation model and our ‘in-hospital’ control design. The ‘in-hospital’ controls are clearly more relevant to the study groups involved than would be a control group in a separate hospital, where the play of environmental changes may be entirely different. However, the design is vulnerable to both Hawthorne effects and contamination. The improvement in the WHO checklist compliance in the control group is suggestive of a contamination effect. Although there was little or no staff transfer between the groups, the control group was aware of the study and the presence of study personnel watching their performance, and this may have induced a type of ‘Hawthorne effect’ in relation to the part of their routine most closely linked in their minds with safety. This may explain why the frequency of attempts at T/O improved in the control group but quality did not. S/O was poorly complied with in both groups; we have previously shown this in all five hospitals that we studied in our wider programme, and concluded that the S/O, as introduced, is not fit for the purpose in a UK environment and should be redesigned.^14^ Conversely, the rise in glitch rate in the active group may represent a Hawthorne effect which faded with time, resulting in better glitch scores before than after the intervention. Most studies of this kind of training have shown positive changes in attitudes and teamwork behaviour, but changes in technical and clinical outcomes have been much less consistently reported.^7^ Clinical endpoints were not suitable primary outcome measures in an intensive study of small numbers of procedures, but the glitch count findings suggest that better teamwork does not necessarily translate into better technical performance.

The use of parallel control groups was the strength of this study, and showed that neither the expected improvement in NOTECHS nor the unexpected deterioration in glitch rate could be attributed to secular trends. Observers could not be blinded, and were therefore aware of the status of the groups during observations although the outcome scales were semiobjective, and this together with the independent scoring method should reduce the bias of the final score. The changes we observed were not those to be expected from observer bias which one would have expected to show opposite changes in glitch count in particular. The study subjects were not selected for representativeness of NHS theatre staff generally, and other groups of staff might have reacted quite differently to the training. The control group in this study was necessarily quite different from the active group in terms of patient characteristics and operations performed. Risk adjustment using statistical techniques might have allowed for the effects of these differences on clinical outcomes if our sample size had been large enough to permit it. The control group, nevertheless, remained an important safeguard against incorrect conclusions based on secular trends, and thereby improved the validity of our findings. Importantly, the clinical outcome data were extracted by clerical staff unaware of the nature of the study. Subjectively, CRM-type training appears to have an effect for a limited period, but we were unable to make any observations which contributed to the question of how often it might need to be repeated. While showing clear benefits in relation to non-technical skills performance and quality of T/O performance, the overall performance of CRM in this study would not commend it as a single strategy for improving patient safety in surgery. A recent systematic review identified generally positive associations between team training and non-technical skills, as we report here, but little evidence of impact on technical performance or patient outcome.^16^ Our findings are therefore consistent with the literature on teamwork training as an isolated intervention, and we suggest that investigation of additional or alternative interventions is therefore merited. Our larger programme of work examines the question of whether interventions focused on team culture (such as CRM), and interventions focused on systems change (such as ‘lean’ quality improvement), may be synergistic in improving staff performance and patient outcomes. In conclusion, we found that CRM-style training was associated with improved non-technical skills performance but not technical performance in operating theatre teams, and that most of the improvement was related to better nurse behaviours. Whether the effect is durable, or can be related to improved patient outcome or cost effectiveness will require further research.

The ‘environment’ category (which includes problems with heating, noise, structural integrity, power and lighting, computer function, other environmental features) represented a small percentage (about 3%) of all glitches in our summary paper on glitches,^10^ and by chance none occurred in this relatively small sample.

## Footnotes

i NHS Trusts are the units of healthcare organisation in NHS England. For the purposes of this study a Trust represents a hospital or a group of hospitals under one management.
